# Comparison and Implications of Mutational Profiles of Myelodysplastic Syndromes, Myeloproliferative Neoplasms, and Myelodysplastic/Myeloproliferative Neoplasms: A Meta-Analysis

**DOI:** 10.3389/fonc.2020.579221

**Published:** 2020-10-07

**Authors:** Ziqi Wan, Bing Han

**Affiliations:** Department of Hematology, Peking Union Medical College Hospital, Chinese Academy of Medical Science, Beijing, China

**Keywords:** myelodysplastic syndromes (MDS), myeloproliferative neoplasms (MPN), myelodysplastic/myeloproliferative neoplasms (MDS/MPN), gene, mutation frequency

## Abstract

Dysplasia and proliferation are histological properties that can be used to diagnose and categorize myeloid tumors in myelodysplastic syndromes (MDS) and myeloproliferative neoplasms (MPN). However, these conditions are not exclusive, and overlap between them leads to another classification, MDS/MPN. As well as phenotype continuity, these three conditions may have genetic relationships that have not yet been identified. This study aimed to obtain their mutational profiles by meta-analysis and explore possible similarities and differences. We reviewed screening studies of gene mutations, published from January 2000 to March 2020, from PubMed and Web of Science. Fifty-three articles were eligible for the meta-analysis, and at most 9,809 cases were involved for any gene. The top mutant genes and their pooled mutation rates were as follows: *SF3B1* (20.2% [95% CI 11.6–30.5%]) in MDS, *TET2* (39.2% [95% CI 21.7–52.0%]) in MDS/MPN, and *JAK2* (67.9% [95% CI 64.1–71.6%]) in MPN. Subgroup analysis revealed that leukemic transformation-related genes were more commonly mutated in high-risk MDS (MDS with multilineage dysplasia and MDS with excess blasts) than that in other MDS entities. Thirteen genes including *ASXL1, U2AF1, SRSF2, SF3B1*, and *ZRSR2* had significantly higher mutation frequencies in primary myelofibrosis (PMF) compared with essential thrombocythemia and polycythemia vera; this difference distinguished PMF from MPN and likened it to MDS. Chronic myelomonocytic leukemia and atypical chronic myeloid leukemia were similar entities but showed several mutational differences. A heat map demonstrated that juvenile myelomonocytic leukemia and MDS/MPN with ring sideroblasts and thrombocytosis were two distinct entities, whereas MDS/MPN-unclassifiable was closest to high-risk MDS. Such genetic closeness or difference reflected features in the pathogenesis, diagnosis, treatment, and progression of these conditions, and could inspire future genetic studies.

## Introduction

Myelodysplastic syndromes (MDS), myeloproliferative neoplasms (MPN), and myelodysplastic/myeloproliferative neoplasms (MDS/MPN) are heterogeneous groups of myeloid tumors, all of which can progress into acute myeloid leukemia (AML) (1). Some distinct entities possess driver genes with more than 90% (or nearly 100%) mutation frequency in total cases. *SF3B1*, for example, is frequently associated with MDS/MPN with ring sideroblasts and thrombocytosis (MDS/MPN-RS-T) (2), and *JAK2* mutation occurs in almost all polycythemia vera (PV) patients (3). However, the mutational profiles of the other entities are less distinct and show different mutation rates within a pool of genes, suggesting both differences and similarities between them. Numerous studies have reported the genetic landscapes of these entities using next-generation sequencing technology, and a handful of literature reviews have summarized each of the three myeloid tumor types separately. However, to the best of our knowledge, no meta-analysis has been conducted to compare their mutational profiles. Therefore, the aim of this study was to determine the gene mutation frequencies of the three myeloid tumor types by meta-analysis, to analyze differences between them, and to gain insight into their relationships and risk factors.

## Methods

This study followed the guidelines for Meta-analysis of Observational Studies (4).

### Systematic Search

Two databases, PubMed and Web of Science, were searched for articles published between January 2000 and March 2020, using the terms “myelodysplastic-myeloproliferative diseases/genetics” in MeSH terms or topic terms; “myelodysplastic syndromes/genetics,” “myeloproliferative disorders/genetics” in MeSH major topic or topic terms; and “sequence analysis” or “sequencing” in titles, abstracts, or topic terms. Keywords “aplastic anemia,” “acute myeloid leukemia,” “paroxysmal nocturnal hemoglobinuria,” “sideroblastic anemia,” “mitochondri^*^,” “drug resistance,” or “drug therapy” in titles and “RNA,” “tool,” “method,” “transplantation,” and “anemia” in topic terms were excluded when searching Web of Science, and the research area was restricted to “hematology.” These restrictions were intended to exclude publications not related to our focus, such as other diseases, tools or method evaluations, drug assessments, and transplantation effects. A manual search was also performed.

Duplicate publications were removed after the initial database search. Case reports, meta-analyses, books, patents, commentaries, reviews, animal studies, and conference-only posters were excluded. Keywords related to transplantation or drug regimens were removed. The remaining articles were then screened by title and abstract, or by full-text review. Studies on single-gene analysis, technique evaluations, other diseases, and other unrelated topics were excluded. Studies involving pediatric patients were excluded to eliminate the potential influence of age. Owing to the distinct Ph+ chromosomal pattern, studies on *BCR-ABL1*-positive chronic myeloid leukemia (CML) were also excluded from this analysis.

Two reviewers (ZW and BH) independently assessed studies for inclusion and extracted the data described below. Disagreements between investigators were resolved by discussion.

### Inclusion Criteria

As randomized controlled trials are not suitable for studying mutational landscapes, we included cohort studies about MDS, MPN, and MDS/MPN or their entities. Inclusion criteria for this meta-analysis were as follows: (1) non-randomized controlled studies; (2) patients diagnosed with MDS, *BCR-ABL1*-negative MPN, MDS/MPN, or their entities; (3) at least six genes sequenced; (4) patient sample size >10; (5) adult patients except for those with juvenile myelomonocytic leukemia (JMML); (6) DNA samples; (7) published between January 2000 and March 2020; (8) contained extractable data, including the names of gene mutations and mutation status of tested patient samples.

If more than one publication involved the same patient population, the most recent study or the one with the largest sample size was selected as the primary study for data extraction. As some studies focused only on triple-negative (*JAK2, CALR, MPL*) patients, which would certainly introduce biases with respect to gene frequencies, such publications were not included in this analysis. To reduce the impact of age on the analysis, adult patient samples were selected, which consequently excluded studies on pediatric patients, although JMML studies were retained in order to obtain a full picture of MDS/MPN. Considering the classification changes to World Health Organization (WHO) criteria in 2008 (5) and 2016 (6), articles about mastocytes and therapy-related myeloid neoplasm were excluded, and chronic myelomonocytic leukemia (CMML) and MDS/MPN-RS-T (formerly known as refractory anemia with ring sideroblasts with thrombocytosis in 2008) were re-classified as MDS/MPN rather than MDS. Genes for which there were fewer than 150 total test cases were excluded from the general myeloid tumor analysis to avoid a false high mutation rate resulting from small sample size.

### Data Extraction

The following data were collected: (1) basic characteristics of the study (first author, publication time, study type, myeloid tumor type and entity, sequencing method, diagnosis criteria); (2) patient characteristics (screened genes, diagnosis, sample sources, ethnicity); and (3) mutation status (gene symbols, mutation numbers, screened gene numbers). To reduce the impact of evolution, we specifically excluded sample data for secondary AML.

We did not use quality scoring for evaluation of the quality of articles, as it is controversial, but conducted subgroup and sensitivity analysis as recommended (4).

### Statistical Analysis

Mutation frequencies, that is, the number of patients carrying mutants as a proportion of all cases screened, were calculated. We ignored different variants of the same gene, recording them as the same gene mutation. Therefore, if one patient carried two or more variants of one gene, we would record this as one mutation. As the mutation frequencies were distributed binomially, rather than following a normal distribution, we applied arcsine transformation to the mutation frequencies to normalize the proportional data. Heterogeneity among studies was examined using Cochrane's Q-test and *I*^2^ measure of inconsistency. Significant heterogeneity was defined as *I*^2^ ≥ 50%. If heterogeneity was considered to be significant, a random-effects model was chosen to obtain the pooled proportions. Otherwise, a fixed-effects model was used to combine the frequencies. The corresponding effect sizes and 95% confidence intervals (CIs) were calculated.

Subgroup analyses were conducted by grouping into specific entities. MDS was separated into low-risk MDS (lMDS) and high-risk MDS (hMDS) groups (7). The lMDS group included MDS with single lineage dysplasia, MDS with ring sideroblasts (MDS-RS), MDS with isolated del(5q) according to the WHO-2016 criteria or refractory cytopenia with unilineage dysplasia, refractory anemia with ring sideroblasts, and MDS with isolated del(5q) according to the WHO-2008 criteria. The hMDS group was composed of MDS with excess blasts and MDS with multilineage dysplasia according to the WHO-2016 criteria, or refractory anemia with excess blasts 1/2 and refractory cytopenia with multilineage dysplasia according to the WHO-2008 criteria. For MDS/MPN, the WHO-2016 classification criteria were adopted, including CMML, JMML, atypical CML (aCML), MDS/MPN-RS-T, and myelodysplastic/myeloproliferative neoplasm-unclassifiable (MDS/MPN-U). MPN was divided into essential thrombocythemia (ET), PV, primary myelofibrosis (PMF), and secondary myelofibrosis (SMF). Two studies (8, 9) have shown that the mutational profile of PMF differs from that of SMF (post-ET MF and post-PV MF). Thus, we excluded data compounding PMF and SMF from the subgroup analysis.

To determine the similarity between entities, clustering analysis was conducted using a 32-gene array. The meta-analyzed mutation frequency was centered and scaled in the aspect of mutation frequency. The distance or dissimilarity was calculated by the Euclidean method. Hierarchical clustering was adopted to obtain possible similarities between entities.

Sensitivity analysis was used to determine the impact of each study on the pooled outcome. Publication bias for genes reported by at least 10 studies was evaluated by Egger's test (significance was defined as *p* < 0.05). All statistical analyses were conducted using the meta-analysis R program (version x64 3.6.3 for Windows).

## Results

### Selection of Studies

In total, 2,417 publications were identified through database searching and 148 duplicate studies were removed. The remaining 2,269 articles were subjected to keyword assessment to remove inappropriate publication types (e.g., case reports, reviews, editorials), incorrect types of study (e.g., animal studies), or articles on unrelated topics (e.g., therapeutic use, drug resistance, transplantation). The 1,428 articles obtained from the database search and 11 studies from the manual search underwent further reviews of title and abstract; 1,358 records were excluded according to the criteria above, and 81 articles were retrieved for full-text evaluation. A total of 53 articles were included in our final analysis after in-depth review (Supplementary Figure 1). The characteristics of the studies are summarized in Supplementary Table 1.

Among these 53 publications, 13, 28, and 14 described studies of patients with MDS, MDS/MPN, and MPN, respectively. Two studies involved both MDS and MDS/MPN, and 47 of the 53 articles considered specific entities.

### Gene Mutation Profiles of MDS, MDS/MPN, and MPN

**Figure 1:**
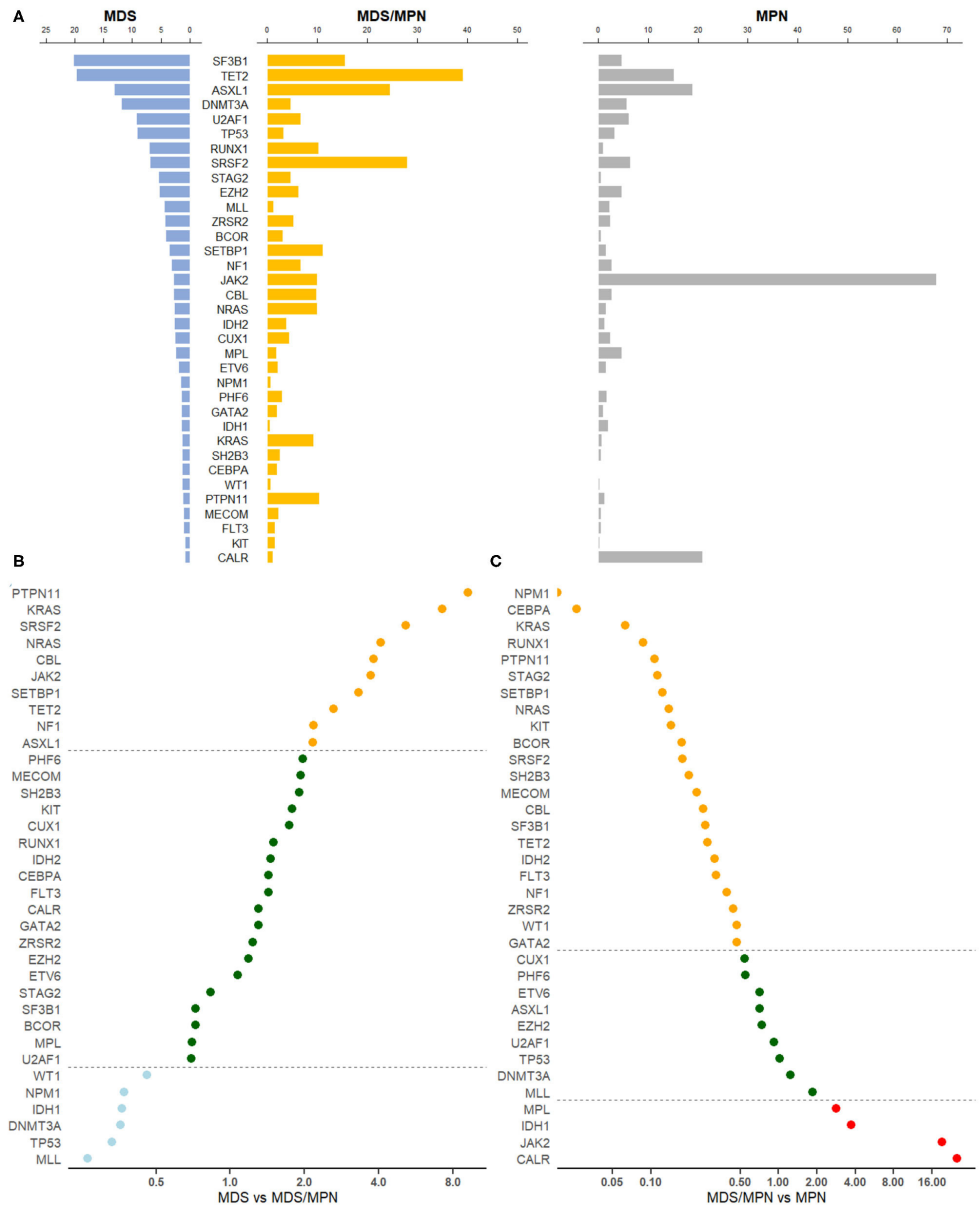
Gene mutation profiles for MDS, MDS/MPN, and MPN. **(A)** Pooled frequencies (%) of major mutations in all three myeloid tumors were plotted. The mutation frequency was obtained by meta-analysis. If heterogeneity was considered to be significant, i.e., *I*^2^ ≥ 50%, a random-effects proportion was used, otherwise a fixed-effects model was adopted to combine the frequencies. Bars in light blue represented mutation frequencies in MDS, orange for MDS/MPN and gray for MPN. **(B)** Odds ratio of meta-mutation-frequency between MDS and MDS/MPN were presented in scatter plots. X-axis was presented in the scale of log_2_. Dots in light blue stood for genes whose odds ratios were <0.5, suggesting higher risk toward MDS, whereas orange dots were representing genes whose odds ratios were more than 2.0, suggesting higher risk toward MDS/MPN. If the odds ratio was in the middle of 0.5 and 2.0, the correspondent gene would be green. The line of ratios, 0.5 and 2.0, were shown horizontally. **(C)** Odds ratio of meta-mutation-frequency between MDS/MPN and MPN are presented in scatter plots. X-axis was presented in the scale of log_2_. Dots in orange stood for genes whose odds ratios were <0.5, suggesting higher risk toward MDS/MPN, whereas red dots were representing genes whose odds ratios were more than 2.0, suggesting higher risk toward MPN. If the odds ratio was in the middle of 0.5 and 2.0, the correspondent gene would be green. The line of ratios, 0.5 and 2.0, were shown horizontally.

After removing genes that were reported by only one study, the mutation profiles of MDS, MDS/MPN, and MPN were analyzed. Collectively, pooled frequencies of 216 genes in MDS, 56 genes in MDS/MPN, and 58 genes in MPN were obtained. These results are presented partially in Figure 1A. *SF3B1, TET2, ASXL1*, and *DNMT3A* were the four genes most often mutated in MDS, with frequencies of 20.2% (95% CI 11.6–30.5%), 19.7% (95% CI 14.9–25.0%), 13.1% (95% CI 8.9–7.9%), and 11.9% [95% CI 10.8–13.1], respectively. In the landscape of MDS/MPN, *TET2, SRSF2, ASXL1, SF3B1* were the four most commonly mutated genes, with frequencies of 39.2% (95% CI 21.7–52.0%), 27.9% (95% CI 20.4–36.2%), 24.6% (16.7–33.1%), and 15.5% (95% CI 5.2–30.1%), respectively. In MPN, the top four genes with their pooled frequencies were: *JAK2* (67.9% [95% CI 64.1–71.6%]), *CALR* (20.9% [95% CI 17.3–24.6%]), *ASXL1* (19.0% [95% CI 12.5–26.4%]), and *TET2* (15.1% [95% CI 12.7–17.8%]). The odds ratios of the mutation frequencies obtained by the meta-analysis are shown in Figures 1B,C: *MLL, TP53, DNMT3A, IDH1, NPM1*, and *WT1* mutations were at least twice as likely to be found in MDS, whereas genes involved in RAS signaling, as well as *SRSF2, JAK2, SETBP1, TET2*, NF1, and *ASXL1*, were found twice as often in MDS/MPN. Three major driver gene mutations, *JAK2, CALR*, and *MPL*, had high frequencies in MPN; however, genes involved in RNA splicing, transcription, and RAS signaling had higher risk of mutation in MDS/MPN compared with MPN.

We further categorized genes by their pathways or functions, based on the classification of Ogawa [(7); Table 1]. RNA splicing gene mutations occurred frequently in MDS and MDS/MPN but less often in MPN. The most significant difference was observed for *SF3B1* mutation, with *p* < 0.0001 for comparisons of both MDS and MDS/MPN with MPN. Although the mutation frequency of *SF3B1* was calculated to be 89.2% (95% CI 84.6–3.1%) in MDS/MPN-RS-T, its proportion in analyzed MDS/MPN cases was low (15%; 202/1319); thus, the mutation rate of *SF3B1* in MDS/MPN was only 15.5% owing to the low mutation frequency in other MDS/MPN entities with low heterogeneity (5.4% [95% CI 4.2–6.8%], *I*^2^ = 26.8%). *TET2*, which is involved in DNA methylation, had a significantly higher mutation frequency in MDS/MPN than in the other two myeloid tumor types (39.2% in MDS/MPN vs. 19.7% in MDS, *p* = 0.0032; 15.1% in MPN, *p* < 0.0001). Genes encoding transcription factors including *BCOR, CEBPA, CUX1, ETV6, GATA2*, and *RUNX1* showed similar profiles in MDS and MDS/MPN but had higher mutation frequencies compared with those in MPN. Notably, *RUNX1* mutation was significantly more frequent in MDS (7.0%, *p* = 0.0001) and MDS/MPN (10.2% *p* < 0.0001) than in MPN, with no significant difference between MDS and MDS/MPN (*p* = 0.1453). MPN was characterized by a high rate of mutations of *JAK2, CALR*, and *MPL*; this group of genes showed similar mutation patterns in MDS and MDS/MPN, except that *JAK2* had a significantly higher mutation frequency in MDS/MPN (9.9 vs. 2.9%, *p* = 0.0059). The *ASXL1* gene, which functions in chromatin modification, was mutated in 24.6% cases of MDS/MPN, significantly higher than the rate of 13.1% in MDS (*p* = 0.0032). The mutation frequencies of genes in the RAS signaling pathway, including *CBL, KRAS, NF1, NRAS*, and *PTPN11*, were all higher in MDS/MPN than in MDS or MPN. The mutation frequency of *TP53* was significantly higher in MDS than in the other tumor types (both *p* < 0.0001), and that of *SETBP1* was significantly higher in MDS/MPN (*p* < 0.0001 for MDS, *p* = 0.0005 for MPN). Cohesion complex gene *STAG2* also showed similar mutation rates in MDS and MDS/MPN, significantly higher than those in MPN (both *p* < 0.0001).

**Table 1:** Gene mutation frequencies (%) and screened cases in MDS, MDS/MPN, and MPN and pathways or functions of genes.

**Pathway/Function**	**Genes**	**MDS**	**MDS/MPN**	**MPN**
RNA splicing	SF3B1	20.22 (3,207)^*^	15.52 (1,319)	4.66 (3,003)
	U2AF1	9.24 (3,085)	6.62 (1,307)	6.17 (2,832)
	SRSF2	6.99 (3,164)	27.94 (1,546)	6.39 (4,083)
	ZRSR2	4.26 (2,703)	5.19 (1,160)	2.35 (2,350)
DNA methylation	TET2	19.71 (3,423)	39.21 (1,512)	15.14 (3,516)
	DNMT3A	11.91 (2,986)	4.62 (1,386)	5.67 (3,516)
	IDH2	2.66 (3,062)	3.84 (1,032)	1.25 (2,952)
	IDH1	1.47 (3,061)	0.54 (834)	1.98 (2,817)
	WT1	1.32 (2,526)	0.61 (249)	0.29 (411)
Transcription	RUNX1	7.02 (3,424)	10.18 (1,694)	0.98 (2,451)
	BCOR	4.14 (2,605)	3.02 (372)	0.54 (2,348)
	CUX1	2.61 (613)	4.44 (492)	2.46 (2,510)
	ETV6	1.93 (2,882)	2.07 (425)	1.49 (449)
	GATA2	1.48 (2,482)	1.92 (156)	0.92 (2,545)
	CEBPA	1.32 (2,389)	1.88 (439)	0.05 (2,067)
Cytokine receptor/	JAK2	2.88 (3,100)	9.93 (1,613)	67.90 (4,089)
tyrosine kinase	MPL	2.45 (2,013)	1.73 (768)	4.75 (3,874)
	FLT3	1.05 (2,341)	1.49 (747)	0.49 (2,168)
	KIT	0.89 (2,055)	1.57 (743)	0.23 (2,306)
	CALR	0.80 (620)	1.04 (641)	20.86 (3,133)
Chromatin	ASXL1	13.08 (3,425)	24.58 (1,961)	18.95 (4,423)
modification	EZH2	5.27 (3,381)	6.17 (1,560)	4.62 (4,246)
RAS signaling	NF1	3.16 (2,162)	6.64 (1,050)	2.71 (2,419)
	CBL	2.77 (3,105)	9.79 (1,964)	2.71 (3,447)
	NRAS	2.66 (2,761)	10.00 (1,777)	1.52 (2,585)
	KRAS	1.39 (2,452)	9.29 (1,675)	0.64 (2,649)
	PTPN11	1.25 (2,451)	10.42 (1,233)	1.23 (2,287)
Others	TP53	9.18 (3,425)	3.24 (553)	3.30 (2,687)
	STAG2	5.50 (2,482)	4.63 (378)	0.54 (2,248)
	MLL	4.40 (613)	1.20 (243)	2.21 (311)
	SETBP1	3.61 (1,104)	11.05 (1,415)	1.51 (2,450)
	NPM1	1.60 (2,602)	0.60 (1,179)	0.00 (203)
	PHF6	1.52 (2,026)	2.95 (420)	1.64 (2,451)
	SH2B3	1.32 (946)	2.48 (229)	0.50 (2,560)
	MECOM	1.14 (613)	2.18 (261)	0.51 (197)

* *Data was presented in the form of gene frequency (%) (cases screened in total)*.

### Subgroup Analysis

**Figure 2:**
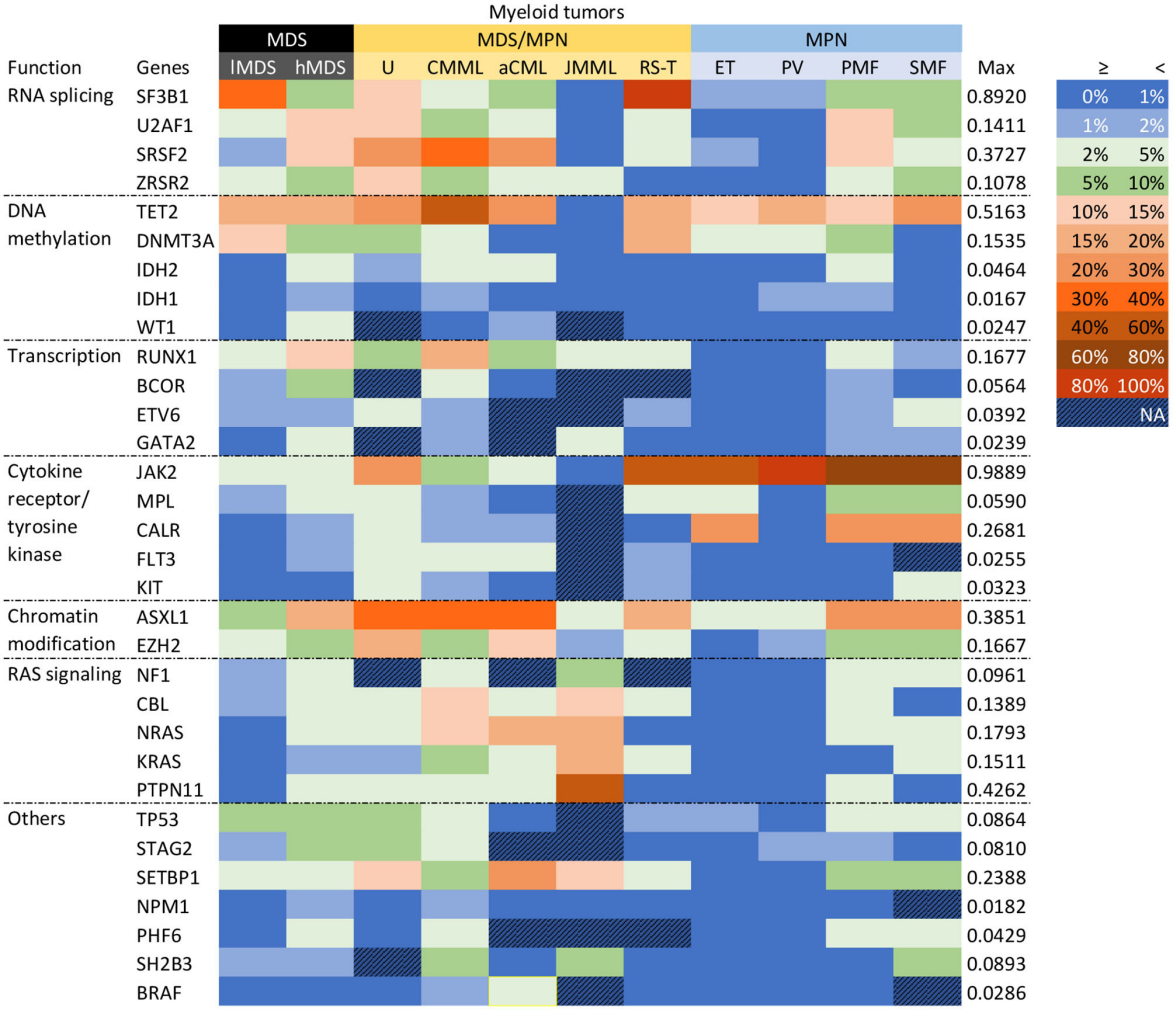
Gene mutational profiles for entities. Genes were clustered according to their functions. Different colors represented different mutation frequency ranges, as shown in the right-hand side. The maximum mutation rate was presented. MDS was subgrouped into low-risk (lMDS) and high-risk (hMDS); MDS/MPN groups were classified depended on WHO-2016 criteria; MPN included ET, PV, PMF, and SMF.

Given that MDS, MDS/MPN, and MPN are all heterogenous myeloid tumors, we conducted subgroup analyses according to their individual entities. The mutational profiles of the various entities are shown in Figure 2. *JAK2* was prominently mutated in PV, with a mutational frequency of 98.9% (95% CI 95.3–100.0%). High frequencies of *JAK2* mutations (>30%) were also seen in MDS/MPN-RS-T, ET, PMF, and SMF. The nine least frequently mutated genes, *IDH2, ETV6, KIT, BRAF, FLT3, WT1, GATA2, NPM1*, and *IDH1*, were all related to AML transformation. Their mutation frequencies in these entities were all lower than 5%.

Within MDS, hMDS had more frequent mutations than lMDS in genes encoding transcription factors (*RUNX1, BCOR, GATA2*), those involved in chromatin modification (*ASXL1, EZH2*) and RAS signaling, and two splicing factor genes (*SRSF2, U2AF1*). On the other hand, *SF3B1* was significantly more likely to be mutated in lMDS, largely because of the 80–90% *SF3B1* mutation rate in MDS-RS. This gene was mutated in 12.7% (95% CI 9.6–16.1%) of cases of other lMDS entities, around twice the rate of the hMDS group (6.6%, 95% CI 5.0–8.5%). Mutation frequencies of DNA methylation genes *TET2* and *DNMT3A* were similar between the two risk groups, but hMDS had more *IDH1, IDH2*, and *WT1* mutations.

Among the MPN entities, *TET2* and *NFE2* were more commonly mutated in PV and SMF than in ET and PMF. The pooled mutation frequency of *TET2* (17.7% [95% CI 14.5–21.1%]) was similar to that in lMDS (17.5% [95% CI 10.1–26.3%]). *ASXL1* had significantly higher mutation frequencies in PMF and SMF than in ET or PV (28.0 and 23.9% vs. 4.0 and 3.3%, all *p* < 0.001; see forest plots in Supplementary Figure 2), with no significant difference between either PMF and SMF (*p* = 0.27) or ET and PV (p = 0.56). Splicing genes *U2AF1, SRSF2, SF3B1*, and *ZRSR2* were also significantly highly mutated in PMF, with frequencies of 12.8, 12.4, 7.3, and 3.5%, respectively. PMF also had high mutation rates of genes including *GNAS, EZH2, SETBP1, NF1, NRAS, MLL*3, *PHF6*, and *RUNX1*. If the data for three MPN driver genes (*JAK2, CALR*, and *MPL*) were excluded, the mutational landscape of PMF was distinct from those of PV and ET. SMF cases showed the highest mutation rates for *TET2, EZH2*, and *ZRSR2* across the four entities, whereas *ASXL1, SF3B1, GNAS, SETBP1, TP53, NRAS*, and *PHF6* had the second highest mutation frequencies in SMF, close to those of PMF. Rates of MPN driver mutations in SMF resembled those in PMF. All these results suggest that PMF and SMF are distinct from PV and ET, although SMF is less so. Data for several genes are listed in Table 2.

**Table 2:** Gene mutation frequencies (%) among MPN entities.

**Genes**	**ET**	**PV**	**PMF**	**SMF**
ASXL1	3.96	3.25	28.00^*^	23.86
U2AF1	0.73	0.00	12.82	6.67
SRSF2	1.83	0.24	12.36	2.28
TET2	10.75	17.71	11.84	23.41
SF3B1	1.71	1.03	7.31	5.74
GNAS	0.15	0.56	7.14	6.45
EZH2	0.88	1.56	5.79	6.78
DNMT3A	3.48	4.26	5.78	0.58
SETBP1	0.00	0.00	5.54	5.21
NF1	0.48	0.89	4.99	2.05
TP53	1.81	0.78	4.96	4.16
NRAS	0.00	0.04	4.68	3.39
MLL3	1.21	0.56	4.65	0.00
PHF6	0.14	0.40	4.29	3.33
ZRSR2	0.23	0.28	3.47	6.40
CBL	0.75	0.89	3.17	0.90
CUX1	0.26	0.18	3.06	5.21
RUNX1	0.14	0.24	3.05	1.06
NFE2	1.22	4.42	1.41	5.08

* *Numbers in red referred to their potential features of a specific gene in a given entity*.

**Figure 3:**
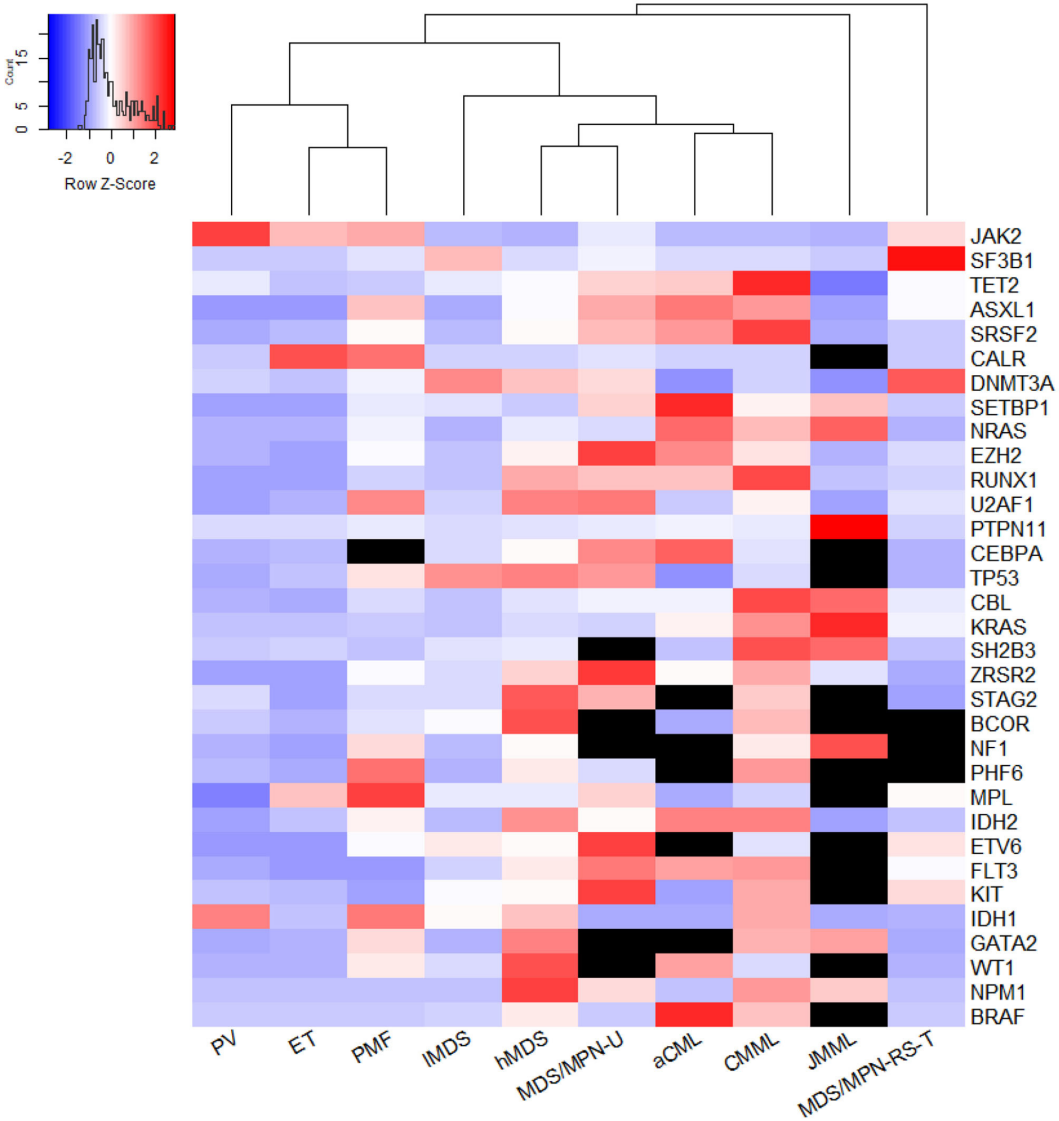
The genetic relationship across entities. The heatmap was plotted using Euclidean distance and hieratical clustering and generated by the method of average agglomeration, using a 32-gene array. The meta-analyzed mutation frequency was centered and scaled in row. Black units stand for unavailable data; redder units represent higher mutation rates while more purple ones mean lower mutation frequency. From this figure, PMF was close to ET. But if excluded the data of three MPN driver genes *JAK2, CALR*, and *MPL*, the mutational landscape of PMF was more distinct from PV and ET (not shown). MDS/MPN-U was computed to be least farther away from high-risk MDS. CMML and aCML were plotted in one tree, which meant CMML was calculated to be least dissimilar from aCML. JMML and MDS/MPN-RS-T were two distinct entities distant from the other three MDS/MPN entities.

Within MDS/MPN, JMML was distinguished by specific mutations in RAS signaling genes with an overall rate of 97.5%. An exception was *SETBP1*, which was mutated in about 10% of JMML cases. In MDS/MPN-RS-T, *SF3B1* had a mutation frequency of 89.2% (95% CI 84.6–93.1%). Otherwise, the pooled mutation proportions for the five DNA methylation genes in MDS/MPN-RS-T were similar to those in lMDS. *JAK2* and *MPL* mutation proportions, however, were similar to those in ET. As shown in the heat map in Figure 3, JMML and MDS/MPN-RS-T were distinct entities, and were clearly different from the other three MDS/MPN entities. CMML had high rates of mutation of *TET2* (51.6% [95% CI 45.0–58.3%]), *SRSF2* (37.3% [95% CI 31.3–43.4%]), *ASXL1* (34.5% [95% CI 28.9–40.3%]), *RUNX1* (16.8% [95% CI 12.6–21.4%]), and *CBL* (13.9% [95% CI 11.8–16.1%]), whereas aCML cases had high rates of mutation of *ASXL1* (38.5% [95% CI 13.0–68.1%]), *TET2* (27.7% [95% CI 14.9–40.3%]), *SRSF2* (25.2% [95% CI 4.2–56.1%]), and *SETBP1* (23.9% [95% CI 14.1–35.3%]). Both CMML and aCML had high mutation rates of *TET2, SRSF2*, and *ASXL1*, partially suggesting the similarity between CMML and aCML. CMML was calculated to be the least dissimilar to aCML; thus, these two entities were plotted in one tree (Figure 3). The frequencies of MPN driver gene mutations were similar in CMML and aCML; however, CMML had higher mutation rates of *TET2, SRSF2, RUNX1, CBL*, and *KRAS*, whereas aCML had more *SETBP1* and *CEBPA* mutations. MDS/MPN-U was found to be closer to hMDS, showing a profile of genes more commonly mutated in hMDS than in lMDS.

### Heterogeneity

Heterogeneity (*I*^2^ ≥ 50%) existed in 86 units (where a unit is one gene mutation in a specific entity) among the 328 units analyzed. Notably, regarding studies screening *SF3B1* mutations in lMDS to omit MDS-RS cases substantially reduced the heterogeneity from 97 to 35%.

### Sensitivity Analysis

Sensitivity analyses of studies screening *ASXL1* in aCML cases and *TET2* in hMDS cases showed that the heterogeneity significantly decreased (from over 90% to 0%) when the studies by Meggendorfer et al. (10) and Papaemmanuil et al. (11) were omitted, indicating that these two studies may have caused the instability of the results. For the other four units with heterogeneity higher than 90%, only two studies were involved in the meta-analysis, suggesting a paucity of data and insufficient number of studies. In total, the great heterogeneity of 20 units out of 86, were caused by insufficient number of studies (only 2 studies involved). Sensitivity analyses revealed that the heterogeneity of 42 units could be reduced to <20% when one study was omitted. The results of the sensitivity analyses are detailed in Supplementary Table 2.

We did additional subgroup analyses for the ethnicity and diagnostic criteria which may contribute to the heterogeneity as well. Out of the 13 mutations analyzed, only the frequencies of *SRSF2* in hMDS and lMDS showed significant difference between Asian and European cohorts, with *p*-value of 0.0074 and 0.0362, respectively. Meanwhile, the proportions of *ASXL1* in lMDS and *SRSF2* in hMDS were significantly different between studies following WHO-2008 and WHO-2016 criteria. But the heterogeneities of all four units were not significantly lowered by the subgrouping. The details of subgroup analyses were listed in Supplementary Table 3.

### Publication Bias

For units eligible for publication bias tests, that is, including at least 10 studies, no significant asymmetry was found (*p* = 0.15–0.95).

## Discussion

This study, to our knowledge, is the first meta-analysis to compare gene mutational profiles across MDS, MDS/MPN, and MPN and demonstrate the continuity among them. We reported the pooled mutation frequency of genes screened in at least 150 cases in the three myeloid tumor types. The order and mutation rates found in MDS were largely identical to those reported by Ogawa (7), except that the mutation percentage of the *SRSF2* gene was between 10 and 15% in their report rather than around 7% according to the current meta-analysis. Our analysis included studies considered in Ogawa's review, and a few additional ones. The results for MDS/MPN and MPN entities were similar to those of reviews by Tanaka and Bejar (12) and Vainchenker and Kralovics (13), respectively.

In the subgroup analysis, the mutation rates of AML transformation-related genes (*IDH2, ETV6, KIT, BRAF, FLT3, WT1, GATA2, NPM1*, and *IDH1*) (14) were lower than 5% for all entities. However, these genes were more frequently mutated in hMDS than in lMDS, reflecting the high risk of leukemic transformation in the hMDS group. Otherwise, the hMDS group displayed higher mutation frequencies of DNA methylation genes, consistent with the fact that hypomethylating agents (HMAs) are first-line therapy for hMDS patients but rarely used for lMDS (15). The lMDS group showed a higher *SF3B1* mutation frequency, indicating the relatively good clinical prognosis of these patients. The mutation rate of *SF3B1* in lMDS when MDS-RS was excluded was still twice that in hMDS. Therefore, high *SF3B1* mutation frequency and low AML transformation-related gene mutation frequency may explain the favorable clinical outcomes in lMDS patients, consistent with the results of studies evaluating the clinical significance of *SF3B1* mutations (16). These genetic features in lMDS support current therapeutic practice, which mainly uses supportive care adapted by personalized genotype, followed by close observation (15).

We separated MDS into lMDS and hMDS following the WHO classification not the International Prognostic Scoring System (IPSS). On the one hand, the WHO classification was used to separate risks apart since a significant difference in AML transformation existed between the two risk groups (7, 17, 18). On the other hand, although the IPSS/IPSS-R score is widely used for assessing prognosis, it is not commonly included in the current publications analyzing gene mutation frequencies. For example, out of 53 publications we analyzed, only 2 had reported the frequencies in each IPSS groups. Indeed, the IPSS classification has been reported to be related to mutation status (19). Thus, if more publications adopt IPSS/IPSS-R classification when analyzing gene mutations, a new meta-analysis is certainly needed to address the relationship between IPSS classification and MDS mutation frequencies.

In MPN, *TET2*, and *NFE2* appeared to be PV-distinct genes based on their significantly higher mutation rates compared with ET and PMF. The results for *NFE2* were consistent with its single gene analyses (20). Elevated *NFE2* levels have been demonstrated to trigger an MPN phenotype in murine models (21, 22), indicating a potential pathophysiological role of *NFE2* in MPN. A recent study showed that *JMJD1C* is a target gene of *NFE2* and participates in a positive feedback loop leading to *NFE2* overexpression (23). However, Tefferi et al. found that *TET2* displayed similar mutation frequencies among MPN subgroups, with no significant impact on survival, leukemic transformation, or thrombosis in PV (24). These different observations were partially due to their small sample size (PV, *n* = 89; ET *n* = 57; PMF, *n* = 60; SMF, *n* = 21) compared with ours (PV, *n* = 510; ET *n* = 1,483; PMF, *n* = 818; SMF, *n* = 126). Tefferi's study also showed a significant difference in *TET2* mutation frequency between PV and ET. *TET2* and *NFE2* mutations were not only common in PV but also abundant in SMF, whose mutation rates were the highest across MPN subgroups. Hence, all these results imply that *TET2* and *NFE2* have functions in the progression of PV.

*ASXL1* and splicing gene mutations were predominantly high in PMF compared with other MPN entities; therefore, they were categorized as PMF-specific genes. This category also included eight more genes identified in our analysis. These PMF-specific genes are commonly present in MDS and MDS/MPN, suggesting that PMF is a mixed myeloproliferative/myelodysplastic syndrome rather than a pure MPN such as PV (13). This assumption was supported by the heat map (not shown) excluding the three main driver gene mutations. However, mutations do not necessarily indicate pathogenetic mechanisms. The assumption on the pathogenetic roles of PMF-specific mutations needs further experiments to verify. *TET2* and *ASXL1* contribute to the pathogenesis of MPN, as demonstrated in zebrafish. Heterozygous loss of *ASXL1* induced an MPN phenotype in half of the zebrafish at the age of 5 months. The exhibition of MPN would be more penetrate if a heterozygous TET2 loss was combined with heterozygous loss of *ASXL1*, whereas a homozygous loss of *TET2* combined with heterozygous *ASXL1* loss leads to AML progression (25). The association of these two genes with AML transformation is further supported by the finding that they are common not only in post-MPN AML but also in *de novo* AML and post-MDS AML (26). Thus, *TET2* and *ASXL1* could be key genes in both the pathogenesis and the progression of MPN. Overall, these mutational features of MPN are reflected in their leukemic transformation risks (10-year risk after diagnosis), which is estimated to be 1% in ET, 4% in PV, and 20% in PMF (27, 28).

Interestingly, SMF exhibited higher mutation rates of PV-distinct genes than did PV itself, but slightly lower rates of PMF-specific mutations than PMF, which may be associated with the pathogenesis of SMF. Compared with PMF, SMF possessed not only a similar frequency of PMF-specific gene mutations but also significantly higher rates of *TET2* mutation. This genetic feature of SMF could explain its higher risk of AML transformation. The 5-year transformation rate after diagnosis is around 18% in SMF but 5% in PMF (8).

Within MDS/MPN, JMML, and MDS/MPN-RS-T had marked gene mutations in RAS signaling and *SF3B1*, respectively. RAS pathway mutations have been traditionally perceived as largely mutually exclusive (29), but this has been disproved by several studies that found 4–17% coexisting genetic hits (30–32). Considering this, our cumulative RAS mutation rate was consistent with those of previous studies (30–32). The leukemic transformation rate of JMML is around 10% over 5 years (32, 33), whereas MDS/MPN-RS-T has a lower rate (34) of two cases out of 82 (2.4%) (35). This difference in leukemic transformation could be partially explained by the difference in genotypes, implying that mutation in RAS signaling might be a negative predictor for AML, whereas *SF3B1* is a favorable indicator. Dysregulation of the RAS signaling pathway is a current hot spot of JMML research with respect to pathogenesis, molecular mechanisms, diagnosis, and therapy (36, 37), as is *SF3B1* mutation in MDS/MPN-RS-T (38–40). MDS/MPN-RS-T showed similar frequencies of mutations of driver genes to ET, and a similar frequency of *SF3B1* mutations to MDS-RS, but it was otherwise distinct from both ET and MDS-RS, indicating its status as a separate entity.

On the other hand, the mutational profiles for CMML and aCML were consistent with the results of their respective reviews (41, 42). Although leukemic transformation rates increased with CMML-specific prognostic model risk levels and varied among reports, its incidence is usually quoted as 15–20% over 3–5 years (41). However, the molecular mechanisms of its pathogenesis and transformation remain unclear. A patient with CMML secondary to familial platelet disorder was reported to have AML transformation owing to a second *CBL* mutation and 11q-acquired uniparental disomy (11q-aUPD) following a previous *RUNX1* mutation, indicating the contribution of *CBL* mutation during the transformation from CMML to AML (43). Later, the role of *CBL* mutation in CMML pathogenesis was investigated in a mouse model. Murine mutant *CBL* not only induced several hallmarks of CMML, for example, sustained myelomonocyte proliferation, but was also related to AML progression in cooperation with the *EVI1* gene (44). These two studies were consistent with our finding that the mutation rate of *CBL* was highest in CMML among all the subgroups analyzed. Owing to hypermethylation caused by loss of *TET2*, HMAs have been approved for CMML since 2004 in the United States. More than half of CMML patients carry this mutation, a higher rate than in any other entity we analyzed. Mutant *TET2* has also been shown to be associated with higher response rates to HMAs in both MDS and CMML patients (45–47).

According to our analysis, CMML was least dissimilar from aCML genetically. Atypical CML cases possessed the same top three mutations as CMML in a different order, and higher rates of *SETBP1* and *CEBPA* mutations. Such genetic likeness or unlikeness may be an indicator of pathogenesis or a predictor of prognosis. *SETBP1*, in particular, could facilitate diagnosis and serve as a negative prognostic factor (48–50), but its precise role in the pathogenesis of aCML remains unclear. The AML progression rate of aCML is around 30–40% according to the literature (42, 51), higher than that of CMML. This difference could be explained by the genetic differences described above; however, more research is needed.

MDS/MPN-U was calculated to be closest to hMDS, reflecting a high risk of leukemic transformation. A clinical trial in 35 MDS/MPN patients, including 14 with MDS/MPN-U, combined azacytidine (an HMA) with ruxolitinib (a JAK1/2-inhibitor) and found better median survival among patients with MDS/MPN-U than those with CMML or aCML (52). Therefore, therapeutic algorithms for MDS/MPN-U could be based on those used for hMDS, with modifications according to patients' genetic profiles, clinical manifestations, and other important factors.

We also reported the heterogeneity of mutation frequency in each entity and analyzed the possible contributions to the high heterogeneity. The results suggested that paucity of data, disturbance of one study, ethnicity of patients and criteria of diagnosis might cause the instability of the results. However, subgrouping by the last two factors did not show significantly low heterogeneity, which might indicate their roles are compounded by other factors we do not include. Another consideration is the insufficiency of data: <150 Asian patients of lMDS were involved to test the role of ethnicity and <200 patients diagnosed by WHO-2016 criteria were included. Therefore, more studies following the newer WHO criteria and reporting different ethnic groups were needed.

In summary, this meta-analysis directly demonstrated the genetic continuity among three myeloid tumor types, MDS, MPN, and MDS/MPN. Distinguishing mutational features were highlighted, and their importance in pathogenesis, diagnosis, prognosis, and therapeutic use were explained and discussed. Our analysis could provide useful insights for future research, including mechanism studies and drug development.

## Data Availability Statement

All datasets presented in this study are included in the article/Supplementary Material.

## Author Contributions

ZW and BH performed the database search, assessed the studies for inclusion, and extracted the data. ZW performed the statistical analyses and wrote the manuscript. BH revised the draft. All authors approved the final submitted version.

## Conflict of Interest

The authors declare that the research was conducted in the absence of any commercial or financial relationships that could be construed as a potential conflict of interest.
