# Supplementary material for: Comparison and Implications of Mutational Profiles of Myelodysplastic Syndromes, Myeloproliferative Neoplasms, and Myelodysplastic/Myeloproliferative Neoplasms: A Meta-Analysis
Source: Front Oncol. 2020 Oct 7;10:579221. doi: 10.3389/fonc.2020.579221 (PMC7575718; doi:10.3389/fonc.2020.579221)
Supplement: Supplementary file 1 [file Data_Sheet_1.pdf]

Supplementary material

Contents

Table S1. Study characteristics for articles included in meta-analysis................2

Table S2. Results of sensitivity analyses..7

Table S3. Results of subgrouping by ethnicity and diagnostic criteria 9

Figure S1. Flow chart eligibility criteria of articles included in meta-analysis ..11

Figure S2. Forest plots of *ASXL1* mutation..12

Figure S3. Heat map excluding the mutation rates of *JAK2, CALR* and *MPL* in MPN entities. 13

Table S1. Study characteristics for articles included in meta-analysis

Study	Myeloid tumor type	Entity availability	Screening method	Diagnosis criteria	DNA Sample sources	Population	Entity included
V. Gelsi-Boyer, 2008[1]	MDS/MPN	Y	PCR, Sanger	WHO-2008, FAB	BM	European	CMML
A. Kohlmann, 2010[2]	MDS/MPN	Y	NGS	WHO-2008	PB, BM	European	CMML
B. Pérez, 2010[3]	MDS/MPN	Y	PCR	IAC	PB, BM	European	JMML
V. Gelsi-Boyer, 2010[4]	MDS/MPN	Y	PCR, Sanger	WHO-2008, FAB	BM	European	CMML
A. M. Jankowska, 2011[5]	MDS/MPN	Y	PCR, Sanger	WHO-2008	BM	American	CMML
K. Yoshida, 2011[6]	MDS, MDS/MPN	Y	NGS	WHO-2008	PB, BM	Asian	8 in MDS, CMML
O. Abdel-Wahab, 2011[7]	MPN	Y	NGS	NA	BM	American	PMF, SMF
O. Abdel-Wahab, 2011*[8]	MPN	Y	NGS	NA	BM	American	PMF, SMF
R. Bejar, 2011[9]	MDS	NA	NGS, Sanger	FAB	BM, OME	American	-
V. Grossmann, 2011[10]	MDS/MPN	Y	NGS	WHO-2008	PB, BM	European	CMML
C. Pérez, 2012[11]	MDS/MPN	Y	Sanger	WHO-2008	PB, BM	European	CMML
M. Brecqueville, 2012[12]	MPN	Y	Sanger	WHO-2008	PB, OME	European	ET, PV, PMF, SMF
M. Meggendorfer, 2012[13]	MDS/MPN	Y	NGS	WHO-2008	PB, BM	European	CMML
A. M. Vannucchi, 2013[14]	MPN	Y	Sanger	WHO-2008	PB, OME	European, American	PMF
E. Papaemmanuil, 2013[15]	MDS, MDS/MPN	Y	NGS	WHO-2008, WHO-2002*	PB, BM	European	7 in MDS, CMML, RS-T
J. Nangalia, 2013[16]	MPN	Y	NGS, Sanger	WHO-2008, BCSH	BM	European	PV, ET, PMF, U
M. Fernandez-Mercado, 2013[17]	MDS	Y	NGS	NA	PB	European	5q-
M. J. Walter, 2013[18]	MDS	NA	NGS	NA	BM	American	-
R. Itzykson, 2013[19]	MDS/MPN	Y	PCR	WHO-2008	PB, BM	European	CMML
R. Piazza, 2013[20]	MDS/MPN	Y	NGS, Sanger	WHO-2008	PB, BM	European	aCML
S. A. Kar, 2013[21]	MDS/MPN	Y	PCR	WHO-2008	BM	American	CMML
V. Gelsi-Boyer, 2013[22]	MDS/MPN	Y	PCR, Sanger	WHO-2008, FAB	BM	European	CMML
A. Tefferi, 2014[23]	MPN	Y	PCR, Sanger	WHO-2008	PB, BM	American	PMF
L. Xu, 2014[24]	MDS	Y	NGS	WHO-2008	BM	Asian	6 in MDS
M. Brecqueville, 2014[25]	MPN	Y	Sanger	WHO-2008	PB, BM	European	PMF, SMF
M. Meggendorfer, 2014[26]	MDS/MPN	Y	NGS	WHO-2008	PB, BM	European	CMML, aCML
P. Lundberg, 2014[27]	MPN	Y	NGS, Sanger	WHO-2008	PB	European	ET, PMF, PV
S. A. Wang, 2014[28]	MDS/MPN	Y	PCR, Sanger	WHO-2008	PB, BM	American	aCML, MDS/MPN-U
T. Haferlach, 2014[29]	MDS	NA	NGS	WHO-2008	PB, BM	European	-

A. Caye, 2015[30]	MDS/MPN	Y	NGS, Sanger	IAC[31]	PB, BM	European	JMML
E. Stieglitz, 2015[32]	MDS/MPN	Y	NGS, Sanger	IAC[31]	PB, BM	American	JMML
S. Jeromin, 2015[33]	MDS/MPN	Y	NGS, Sanger	WHO-2008	BM	European	RS-T
C. C. Mason, 2016[34]	MDS/MPN	Y	NGS	WHO-2008	PB, BM	American	CMML
C. Elena, 2016[35]	MDS/MPN	Y	NGS	WHO-2016, FAB	PB, BM	European	CMML
G. Rotunno, 2016[36]	MPN	Y	NGS, Sanger, PCR	IWG-MRT	PB	European	SMF
L. Palomo, 2016[37]	MDS/MPN	Y	NGS	WHO-2008, FAB	PB, BM	European	CMML
M. M. Patnaik, 2016[38]	MDS/MPN	Y	NGS	WHO-2008	BM	American	RS-T
S. Delic, 2016[39]	MPN	Y	NGS	WHO-2008	PB, BM	European	ET, PMF, PV
A. Tefferi, 2017[40]	MDS	NA	NGS	WHO-2008	BM	American	-
B. J. Patel, 2017[41]	MDS/MPN	Y	PCR, Sanger	WHO-2008	PB, BM	American	CMML
F. Xu, 2017[42]	MDS	NA	NGS	WHO-2008	BM, OME	Asian	-
I. Martín, 2017[43]	MDS	Y	NGS	WHO-2016	BM	European	MDS-RS
J. Song, 2017[44]	MPN	NA	NGS	WHO-2008	PB, BM	American	-
M. M. Patnaik, 2017[45]	MDS/MPN	Y	NGS	WHO-2008, 2016	BM	American	aCML
M. Meggendorfer, 2017[46]	MDS	Y	NGS	WHO-2008, 2016	PB, BM	European	5q-
S. Y. Kim, 2017[47]	MDS	Y	NGS	WHO-2008	BM	Asian	5 in MDS
J. Grinfeld, 2018[48]	MPN	Y	NGS	WHO-2016, BCSH	PB, BM, WB	European	ET, MF, PV, MPN-U
M. Meggendorfer, 2018[49]	MDS/MPN	Y	NGS, Sanger	WHO-2016	PB, BM	European	4 in MDS
N. Murakami, 2018[50]	MDS/MPN	Y	NGS	IAC[51]	PB, BM	Asian	JMML
P. Bose, 2018[52]	MDS/MPN	Y	NGS	WHO-2008	PB, BM	American	CMML, MDS/MPN-U
H. Gill, 2019[53]	MPN	Y	NGS	WHO-2016, IWG-MRT[54]	BM	Asian	PMF, SMF
F. Courtier, 2020[55]	MPN	Y	NGS	WHO-2008	PB, BM	European	PMF, SMF
L. Jiang, 2020[56]	MDS	Y	NGS	WHO-2016	BM	Asian	7 in MDS

Abbreviations: PB, peripheral blood; BM, bone marrow smears; OME, oral mucosa epithelia; WB, whole blood; BCSH, British committee of standards in haematology; IAC, internationally accepted criteria; IWG-MRT, International Working Group for Myeloproliferative neoplasms Research and Treatment;

*RCMD-RS were retained as a separate category

1. Gelsi-Boyer V, Trouplin V, Adélaïde J, et al. Genome profiling of chronic myelomonocytic leukemia: frequent alterations of RAS and RUNX1 genes. *BMC Cancer.* 2008;8:299-299.
2. Kohlmann A, Grossmann V, Klein H-U, et al. Next-generation sequencing technology reveals a characteristic pattern of molecular mutations in 72.8% of chronic myelomonocytic leukemia by detecting frequent alterations in TET2, CBL, RAS, and RUNX1. *J Clin Oncol.* 2010;28(24):3858-3865.
3. Pérez B, Kosmider O, Cassinat B, et al. Genetic typing of CBL, ASXL1, RUNX1, TET2 and JAK2 in juvenile myelomonocytic leukaemia reveals a genetic profile distinct from chronic myelomonocytic leukaemia. *Br J Haematol.* 2010;151(5):460-468.
4. Gelsi-Boyer V, Trouplin V, Roquain J, et al. ASXL1 mutation is associated with poor prognosis and acute transformation in chronic myelomonocytic leukaemia. *Br J Haematol.* 2010;151(4):365-375.
5. Jankowska AM, Makishima H, Tiu RV, et al. Mutational spectrum analysis of chronic myelomonocytic leukemia includes genes associated with epigenetic regulation: UTX, EZH2, and DNMT3A. *Blood.* 2011;118(14):3932-3941.
6. Yoshida K, Sanada M, Shiraishi Y, et al. Frequent pathway mutations of splicing machinery in myelodysplasia. *Nature.* 2011;478(7367):64-69.
7. Abdel-Wahab O, Pardanani A, Patel J, et al. Concomitant analysis of EZH2 and ASXL1 mutations in myelofibrosis, chronic myelomonocytic leukemia and blast-phase myeloproliferative neoplasms. *Leukemia.* 2011;25(7):1200-1202.
8. Abdel-Wahab O, Pardanani A, Rampal R, Lasho TL, Levine RL, Tefferi A. DNMT3A mutational analysis in primary myelofibrosis, chronic myelomonocytic leukemia and advanced phases of myeloproliferative neoplasms. *Leukemia.* 2011;25(7):1219-1220.
9. Bejar R, Stevenson K, Abdel-Wahab O, et al. Clinical effect of point mutations in myelodysplastic syndromes. *N Engl J Med.* 2011;364(26):2496-2506.
10. Grossmann V, Kohlmann A, Eder C, et al. Molecular profiling of chronic myelomonocytic leukemia reveals diverse mutations in >80% of patients with TET2 and EZH2 being of high prognostic relevance. *Leukemia.* 2011;25(5):877-879.
11. Pérez C, Martínez-Calle N, Martín-Subero JI, et al. TET2 mutations are associated with specific 5-methylcytosine and 5-hydroxymethylcytosine profiles in patients with chronic myelomonocytic leukemia. *PLoS One.* 2012;7(2):e31605-e31605.
12. Brecqueville M, Rey J, Bertucci F, et al. Mutation analysis of ASXL1, CBL, DNMT3A, IDH1, IDH2, JAK2, MPL, NF1, SF3B1, SUZ12, and TET2 in myeloproliferative neoplasms. *Genes Chromosomes Cancer.* 2012;51(8):743-755.
13. Meggendorfer M, Roller A, Haferlach T, et al. SRSF2 mutations in 275 cases with chronic myelomonocytic leukemia (CMML). *Blood.* 2012;120(15):3080-3088.
14. Vannucchi AM, Lasho TL, Guglielmelli P, et al. Mutations and prognosis in primary myelofibrosis. *Leukemia.* 2013;27(9):1861-1869.
15. Papaemmanuil E, Gerstung M, Malcovati L, et al. Clinical and biological implications of driver mutations in myelodysplastic syndromes. *Blood.* 2013;122(22):3616-3699.
16. Nangalia J, Massie CE, Baxter EJ, et al. Somatic CALR mutations in myeloproliferative neoplasms with nonmutated JAK2. *N Engl J Med.* 2013;369(25):2391-2405.
17. Fernandez-Mercado M, Burns A, Pellagatti A, et al. Targeted re-sequencing analysis of 25 genes commonly mutated in myeloid disorders in del(5q) myelodysplastic syndromes. *Haematologica.* 2013;98(12):1856-1864.
18. Walter MJ, Shen D, Shao J, et al. Clonal diversity of recurrently mutated genes in myelodysplastic syndromes. *Leukemia.* 2013;27(6):1275-1282.
19. Itzykson R, Kosmider O, Renneville A, et al. Clonal architecture of chronic myelomonocytic leukemias. *Blood.* 2013;121(12):2186-2198.
20. Piazza R, Valletta S, Winkelmann N, et al. Recurrent SETBP1 mutations in atypical chronic myeloid leukemia. *Nat Genet.* 2013;45(1):18-24.
21. Kar SA, Jankowska A, Makishima H, et al. Spliceosomal gene mutations are frequent events in the diverse mutational spectrum of chronic myelomonocytic leukemia but largely absent in juvenile myelomonocytic leukemia. *Haematologica.* 2013;98(1):107-113.
22. Gelsi-Boyer V, Cervera N, Bertucci F, et al. Molecular similarity between myelodysplastic form of chronic myelomonocytic leukemia and refractory anemia with ring sideroblasts. *Haematologica.* 2013;98(4):576-583.
23. Tefferi A, Lasho TL, Finke CM, et al. CALR vs JAK2 vs MPL-mutated or triple-negative myelofibrosis: clinical, cytogenetic and molecular comparisons. *Leukemia.* 2014;28(7):1472-1477.
24. Xu L, Gu Z-H, Li Y, et al. Genomic landscape of CD34+ hematopoietic cells in myelodysplastic syndrome

and gene mutation profiles as prognostic markers. *Proc Natl Acad Sci U S A.* 2014;111(23):8589-8594.
25. Brecqueville M, Rey J, Devillier R, et al. Array comparative genomic hybridization and sequencing of 23 genes in 80 patients with myelofibrosis at chronic or acute phase. *Haematologica.* 2014;99(1):37-45.
26. Meggendorfer M, Haferlach T, Alpermann T, et al. Specific molecular mutation patterns delineate chronic neutrophilic leukemia, atypical chronic myeloid leukemia, and chronic myelomonocytic leukemia. *Haematologica.* 2014;99(12):e244-246.
27. Lundberg P, Karow A, Nienhold R, et al. Clonal evolution and clinical correlates of somatic mutations in myeloproliferative neoplasms. *Blood.* 2014;123(14):2220-2228.
28. Wang SA, Hasserjian RP, Fox PS, et al. Atypical chronic myeloid leukemia is clinically distinct from unclassifiable myelodysplastic/myeloproliferative neoplasms. *Blood.* 2014;123(17):2645-2651.
29. Haferlach T, Nagata Y, Grossmann V, et al. Landscape of genetic lesions in 944 patients with myelodysplastic syndromes. *Leukemia.* 2014;28(2):241-247.
30. Caye A, Strullu M, Guidez F, et al. Juvenile myelomonocytic leukemia displays mutations in components of the RAS pathway and the PRC2 network. *Nat Genet.* 2015;47(11):1334-1340.
31. Chan RJ, Cooper T, Kratz CP, Weiss B, Loh ML. Juvenile myelomonocytic leukemia: a report from the 2nd International JMML Symposium. *Leuk Res.* 2009;33(3):355-362.
32. Stieglitz E, Taylor-Weiner AN, Chang TY, et al. The genomic landscape of juvenile myelomonocytic leukemia. *Nat Genet.* 2015;47(11):1326-1333.
33. Jeromin S, Haferlach T, Weissmann S, et al. Refractory anemia with ring sideroblasts and marked thrombocytosis cases harbor mutations in SF3B1 or other spliceosome genes accompanied by JAK2V617F and ASXL1 mutations. *Haematologica.* 2015;100(4):e125-127.
34. Mason CC, Khorashad JS, Tantravahi SK, et al. Age-related mutations and chronic myelomonocytic leukemia. *Leukemia.* 2016;30(4):906-913.
35. Elena C, Galli A, Such E, et al. Integrating clinical features and genetic lesions in the risk assessment of patients with chronic myelomonocytic leukemia. *Blood.* 2016;128(10):1408-1417.
36. Rotunno G, Pacilli A, Artusi V, et al. Epidemiology and clinical relevance of mutations in postpolycythemia vera and postessential thrombocythemia myelofibrosis: A study on 359 patients of the AGIMM group. *Am J Hematol.* 2016;91(7):681-686.
37. Palomo L, Garcia O, Arnan M, et al. Targeted deep sequencing improves outcome stratification in chronic myelomonocytic leukemia with low risk cytogenetic features. *Oncotarget.* 2016;7(35):57021-57035.
38. Patnaik MM, Lasho TL, Finke CM, et al. Predictors of survival in refractory anemia with ring sideroblasts and thrombocytosis (RARS-T) and the role of next-generation sequencing. *Am J Hematol.* 2016;91(5):492-498.
39. Delic S, Rose D, Kern W, et al. Application of an NGS-based 28-gene panel in myeloproliferative neoplasms reveals distinct mutation patterns in essential thrombocythaemia, primary myelofibrosis and polycythaemia vera. *Br J Haematol.* 2016;175(3):419-426.
40. Tefferi A, Lasho TL, Patnaik MM, et al. Targeted next-generation sequencing in myelodysplastic syndromes and prognostic interaction between mutations and IPSS-R. *Am J Hematol.* 2017;92(12):1311-1317.
41. Patel BJ, Przychodzen B, Thota S, et al. Genomic determinants of chronic myelomonocytic leukemia. *Leukemia.* 2017;31(12):2815-2823.
42. Xu F, Wu L-Y, He Q, et al. Exploration of the role of gene mutations in myelodysplastic syndromes through a sequencing design involving a small number of target genes. *Sci Rep.* 2017;7:43113-43113.
43. Martín I, Such E, Navarro B, et al. Prognostic impact of gene mutations in myelodysplastic syndromes with ring sideroblasts. *Blood Cancer J.* 2017;7(12):630-630.
44. Song J, Hussaini M, Zhang H, et al. Comparison of the Mutational Profiles of Primary Myelofibrosis, Polycythemia Vera, and Essential Thrombocytosis. *Am J Clin Pathol.* 2017;147(5):444-452.
45. Patnaik MM, Barraco D, Lasho TL, et al. Targeted next generation sequencing and identification of risk factors in World Health Organization defined atypical chronic myeloid leukemia. *Am J Hematol.* 2017;92(6):542-548.
46. Meggendorfer M, Haferlach C, Kern W, Haferlach T. Molecular analysis of myelodysplastic syndrome with isolated deletion of the long arm of chromosome 5 reveals a specific spectrum of molecular mutations with prognostic impact: a study on 123 patients and 27 genes. *Haematologica.* 2017;102(9):1502-1510.
47. Kim SY, Kim K, Hwang B, et al. The high frequency of the U2AF1 S34Y mutation and its association with isolated trisomy 8 in myelodysplastic syndrome in Asians, but not in Caucasians. *Leuk Res.* 2017;61:96-103.
48. Grinfeld J, Nangalia J, Baxter EJ, et al. Classification and Personalized Prognosis in Myeloproliferative

Neoplasms. *N Engl J Med.* 2018;379(15):1416-1430.
49. Meggendorfer M, Jeromin S, Haferlach C, Kern W, Haferlach T. The mutational landscape of 18 investigated genes clearly separates four subtypes of myelodysplastic/myeloproliferative neoplasms. *Haematologica.* 2018;103(5):e192-e195.
50. Murakami N, Okuno Y, Yoshida K, et al. Integrated molecular profiling of juvenile myelomonocytic leukemia. *Blood.* 2018;131(14):1576-1586.
51. Pinkel D. Differentiating juvenile myelomonocytic leukemia from infectious disease. *Blood.* 1998;91(1):365-367.
52. Bose P, Nazha A, Komrokji RS, et al. Mutational landscape of myelodysplastic/myeloproliferative neoplasm-unclassifiable. *Blood.* 2018;132(19):2100-2103.
53. Gill H, Ip H-W, Yim R, et al. Next-generation sequencing with a 54-gene panel identified unique mutational profile and prognostic markers in Chinese patients with myelofibrosis. *Ann Hematol.* 2019;98(4):869-879.
54. Barosi G, Mesa RA, Thiele J, et al. Proposed criteria for the diagnosis of post-polycythemia vera and post-essential thrombocythemia myelofibrosis: a consensus statement from the International Working Group for Myelofibrosis Research and Treatment. *Leukemia.* 2008;22(2):437-438.
55. Courtier F, Garnier S, Carbuccia N, et al. Targeted molecular characterization shows differences between primary and secondary myelofibrosis. *Genes, Chromosomes and Cancer.* 2020;59(1):30-39.
56. Jiang L, Luo Y, Zhu S, et al. Mutation status and burden can improve prognostic prediction of patients with lower-risk myelodysplastic syndromes. *Cancer Sci.* 2020;111(2):580-591.

Table S2. Results of sensitivity analyses

Genes	Frequency	Entities	I^2 (%)	Results of sensitivity analyses	After-omitting I^2 (%)
SF3B1	0.3855	lMDS	96.9	-	-
KIT	0.0080	hMDS	92.8	Insufficient data	-
TP53	0.0416	SMF	92.4	Insufficient data	-
ASXL1	0.3851	aCML	92.1	Omitting M. Meggendorfer, 2014	0.0
SETBP1	0.0325	RS-T	91.8	Insufficient data	-
TET2	0.1931	hMDS	91.7	Omitting E. Papaemmanuil, 2013	0.0
NRAS	0.0271	U	91.3	Insufficient data	-
SETBP1	0.1174	JMML	88.8	Omitting E. Stieglitz, 2015	0.0
PHF6	0.0209	hMDS	88.3	Insufficient data	-
SRSF2	0.2522	aCML	86.7	Insufficient data	-
CEBPA	0.0111	hMDS	86.4	Omitting E. Papaemmanuil, 2013	0.0
SRSF2	0.1152	hMDS	86.2	-	-
TET2	0.1746	lMDS	82.9	-	-
CALR	0.2236	SMF	82.7	Omitting G. Rotunno, 2016	0.0
EZH2	0.0396	RS-T	82.1	Omitting M. M. Patnaik, 2016	0.0
RUNX1	0.0923	aCML	81.6	Omitting R. Piazza, 2013	0.0
JAK2	0.4147	RS-T	81.5	-	-
TP53	0.0818	lMDS	80.7	Omitting M. Meggendorfer, 2017	56.2
U2AF1	0.0233	aCML	80.6	Insufficient data	-
DNMT3A	0.0348	ET	79.9	Omitting M. Brecqueville, 2012	0.4
JAK2	0.9889	PV	78.5	Omitting P. Lundberg, 2014	0.0
ASXL1	0.2800	PMF	78.2	-	-
ASXL1	0.0593	lMDS	78.0	-	-
DNMT3A	0.0096	aCML	77.2	Omitting M. Meggendorfer, 2018	0.0
NF1	0.0373	hMDS	76.9	Omitting L. Xu, 2014	0.0
NRAS	0.1717	aCML	75.8	Omitting M. Meggendorfer, 2018	4.0
ZRSR2	0.0347	PMF	75.5	Insufficient data	-
DNMT3A	0.0089	JMML	74.9	Insufficient data	-
TP53	0.0864	hMDS	74.6	Omitting S. Y. Kim, 2017	47.1
IDH1	0.0167	PMF	74.4	-	-
SRSF2	0.3727	CMML	74.3	Omitting P. Bose, 2018	50.7
CBL	0.0440	aCML	73.8	Omitting M. M. Patnaik, 2017	0.0
KRAS	0.0106	U	73.6	Insufficient data	-
RUNX1	0.0305	PMF	73.3	Omitting S. Delic, 2016	45.7
EZH2	0.0678	SMF	72.3	Omitting O. Abdel-Wahab, 2011	51.2
JAK2	0.0240	hMDS	72.2	Omitting L. Xu, 2014	19.9
TET2	0.2770	aCML	71.4	Omitting M. Meggendorfer, 2014	0.0
TET2	0.5163	CMML	70.6	-	-
MPL	0.0146	CMML	70.4	Omitting C. C. Mason, 2016	54.0
JAK2	0.0309	aCML	70.2	Omitting R. Piazza, 2013	0.0
FLT3	0.0210	aCML	70.0	Omitting R. Piazza, 2013	0.0
CUX1	0.0306	PMF	69.6	Omitting P. Lundberg, 2014	0.0
IDH1	0.0111	hMDS	68.6	-	-
CUX1	0.0018	PV	68.5	Insufficient data	-
ASXL1	0.3447	CMML	68.5	-	-
RUNX1	0.1677	CMML	68.4	Omitting C. Elena, 2016	12.9
CBL	0.0317	PMF	68.2	-	-

SRSF2	0.0165	lMDS	67.8	-	-
TP53	0.0181	ET	67.6	-	-
NOTCH1	0.0300	hMDS	67.2	Insufficient data	-
ASXL1	0.0407	JMML	67.0	Omitting N. Murakami, 2018	0.0
CUX1	0.0452	CMML	67.0	Omitting E. Papaemmanuil, 2013	0.0
FLT3	0.0106	RS-T	66.8	Insufficient data	-
KIT	0.0106	RS-T	66.8	Insufficient data	-
SRSF2	0.1236	PMF	66.4	Omitting S. Delic, 2016	0.0
WT1	0.0247	hMDS	65.6	Omitting S. Y. Kim, 2017	0.0
NPM1	0.0074	U	65.2	Omitting P. Bose, 2018	0.0
MPL	0.0340	ET	65.0	Omitting M. Brecqueville, 2012	0.0
CEBPA	0.0344	aCML	64.5	Omitting M. M. Patnaik, 2017	0.0
ASXL1	0.1844	hMDS	64.4	Omitting S. Y. Kim, 2017	15.0
SRSF2	0.0183	ET	64.2	Insufficient data	-
GATA2	0.0106	SMF	64.1	Insufficient data	-
RUNX1	0.0106	SMF	64.1	Insufficient data	-
U2AF1	0.1282	PMF	63.7	Omitting H. Gill, 2019	0.0
NF1	0.0961	JMML	63.7	Omitting E. Stieglitz, 2015	44.6
IDH2	0.0066	RS-T	63.5	Omitting S. Jeromin, 2015	0.0
FLT3	0.0224	CMML	63.4	Omitting C. C. Mason, 2016	0.0
TET2	0.2341	SMF	62.9	Omitting F. Courtier, 2020	0.0
EZH2	0.0146	JMML	62.5	Omitting S. A. Kar, 2013	7.9
NOTCH1	0.0063	SMF	62.3	Insufficient data	-
CBL	0.0090	SMF	62.1	Omitting F. Courtier, 2020	0.0
TET2	0.1939	RS-T	62.0	Omitting M. M. Patnaik, 2016	32.1
ETV6	0.0165	lMDS	61.7	Omitting M. Meggendorfer, 2017	0.0
JAK2	0.7366	SMF	59.5	Omitting H. Gill, 2019	19.6
MPL	0.0502	SMF	58.4	Omitting G. Rotunno, 2016	21.6
U2AF1	0.0302	lMDS	57.8	Omitting S. Y. Kim, 2017	27.5
NF1	0.0205	SMF	57.3	Insufficient data	-
SF3B1	0.0575	aCML	57.2	Omitting R. Piazza, 2013	0.0
IDH2	0.0053	lMDS	56.7	Omitting M. Meggendorfer, 2017	33.2
SETBP1	0.2388	aCML	56.6	Omitting M. M. Patnaik, 2017	10.9
DNMT3A	0.0058	SMF	53.8	Omitting F. Courtier, 2020	0.0
CALR	0.0112	CMML	53.7	Omitting M. Meggendorfer, 2018	0.0
RUNX1	0.0938	U	52.5	Insufficient data	-
JAK2	0.0550	CMML	52.0	Omitting V. Gelsi-Boyer, 2013	0.0
PHF6	0.0032	lMDS	51.8	Omitting M. Meggendorfer, 2017	0.0
IDH1	0.0159	PV	51.2	Omitting J. Grinfeld, 2018	0.0

Table S3. Results of subgrouping by ethnicity and diagnostic criteria

Gene	Pooled Mutation Frequency in Entity	Entity	I^2(%)	Ethnicity	I^2	Proportion	P-value	Diagnostic criteria	I^2	Proportion	P-value
SF3B1	0.3855	lMDS	96.9	Asian	88.90%	0.4143 [0.1676; 0.6867]		WHO-2008	89.00%	0.4085 [0.2014; 0.6342]	
				European	98.50%	0.3536 [0.0750; 0.7047]	0.7875	WHO-2016	97.20%	0.6487 [0.1604; 0.9879]	0.4048
SRSF2	0.1152	hMDS	86.2	Asian	74.70%	0.0957 [0.0508; 0.1528]		WHO-2008	85.50%	0.1329 [0.0720; 0.2087]	
				European	--	0.1945 [0.1513; 0.2418]	0.0074	WHO-2016	--	0.0536 [0.0197; 0.1027]	0.0464
TET2	0.1746	lMDS	82.9	Asian	56.20%	0.1228 [0.0331; 0.2581]		WHO-2008	79.40%	0.1941 [0.0998; 0.3104]	
				European	86.70%	0.2038 [0.1137; 0.3121]	0.3137	WHO-2016	94.20%	0.1598 [0.0022; 0.4904]	0.8208
JAK2	0.4147	RS-T	81.5	American	--	0.3333 [0.2084; 0.4715]		WHO-2008	87.40%	0.3738 [0.1608; 0.6165]	
				European	82.00%	0.4418 [0.2455; 0.6480]	0.3857	WHO-2016	--	0.5111 [0.3667; 0.6546]	0.3407
ASXL1	0.2800	PMF	78.2	American	91.00%	0.2149 [0.0670; 0.4173]					
				European	76.60%	0.3190 [0.1953; 0.4573]	0.3775[1]				
				Asian	--	0.2857 [0.1867; 0.3964]	--	WHO-2008	77.50%	0.3056 [0.2351; 0.3810]	
				European, American	--	0.2493 [0.2193; 0.2807]	0.6615[2]	WHO-2016	--	0.2857 [0.1867; 0.3964]	0.7631
ASXL1	0.0593	lMDS	78	Asian	81.10%	0.0530 [0.0000; 0.2298]		WHO-2008	66.00%	0.0797 [0.0327; 0.1450]	
				European	81.90%	0.0577 [0.0184; 0.1169]	0.944	WHO-2016	0.00%	0.0114 [0.0009; 0.0333]	0.0067
IDH1	0.0167	PMF	74.4	All European				All WHO-2008			
TET2	0.5163	CMML	70.6	American	0.00%	0.4707 [0.4152; 0.5265]		WHO-2008	69.00%	0.5275 [0.4532; 0.6011]	
				European	81.00%	0.5538 [0.4456; 0.6594]	0.1812	WHO-2016	--	0.4439 [0.3781; 0.5108]	0.1012

IDH1	0.0111	hMDS	68.6	Asian	73.90%	0.0079 [0.0000; 0.0354]		WHO-2008	68.70%	0.0066 [0.0000; 0.0251]	
				European	--	0.0205 [0.0074; 0.0398]	0.3551	WHO-2016	--	0.0357 [0.0095; 0.0778]	0.0782
ASXL1	0.3447	CMML	68.5	American	41.30%	0.3042 [0.2370; 0.3759]		WHO-2008	71.90%	0.3400 [0.2742; 0.4090]	
				European	69.10%	0.3713 [0.3001; 0.4454]	0.1931	WHO-2016	--	0.3692 [0.3059; 0.4348]	0.5409
CBL	0.0317	PMF	68.2	Asian	--	0.0286 [0.0028; 0.0801]					
				European	78.30%	0.0257 [0.0002; 0.0924]		WHO-2008	73.00%	0.0313 [0.0066; 0.0734]	
				European, American	--	0.0435 [0.0271; 0.0635]	0.7015	WHO-2016	--	0.0286 [0.0028; 0.0801]	0.9184
SRSF2	0.0165	lMDS	67.8	Asian	0.00%	0.0028 [0.0000; 0.0198]		WHO-2008	76.20%	0.0080 [0.0000; 0.0415]	
				European	73.90%	0.0347 [0.0095; 0.0746]	0.0362	WHO-2016	0.00%	0.0419 [0.0167; 0.0777]	0.0997
TP53	0.0181	ET	67.6					WHO-2008	83.30%	0.0160 [0.0000; 0.1274]	
				All European				WHO-2016	--	0.0174 [0.0111; 0.0252]	0.9651

1 American vs European; 2 Among all four groups

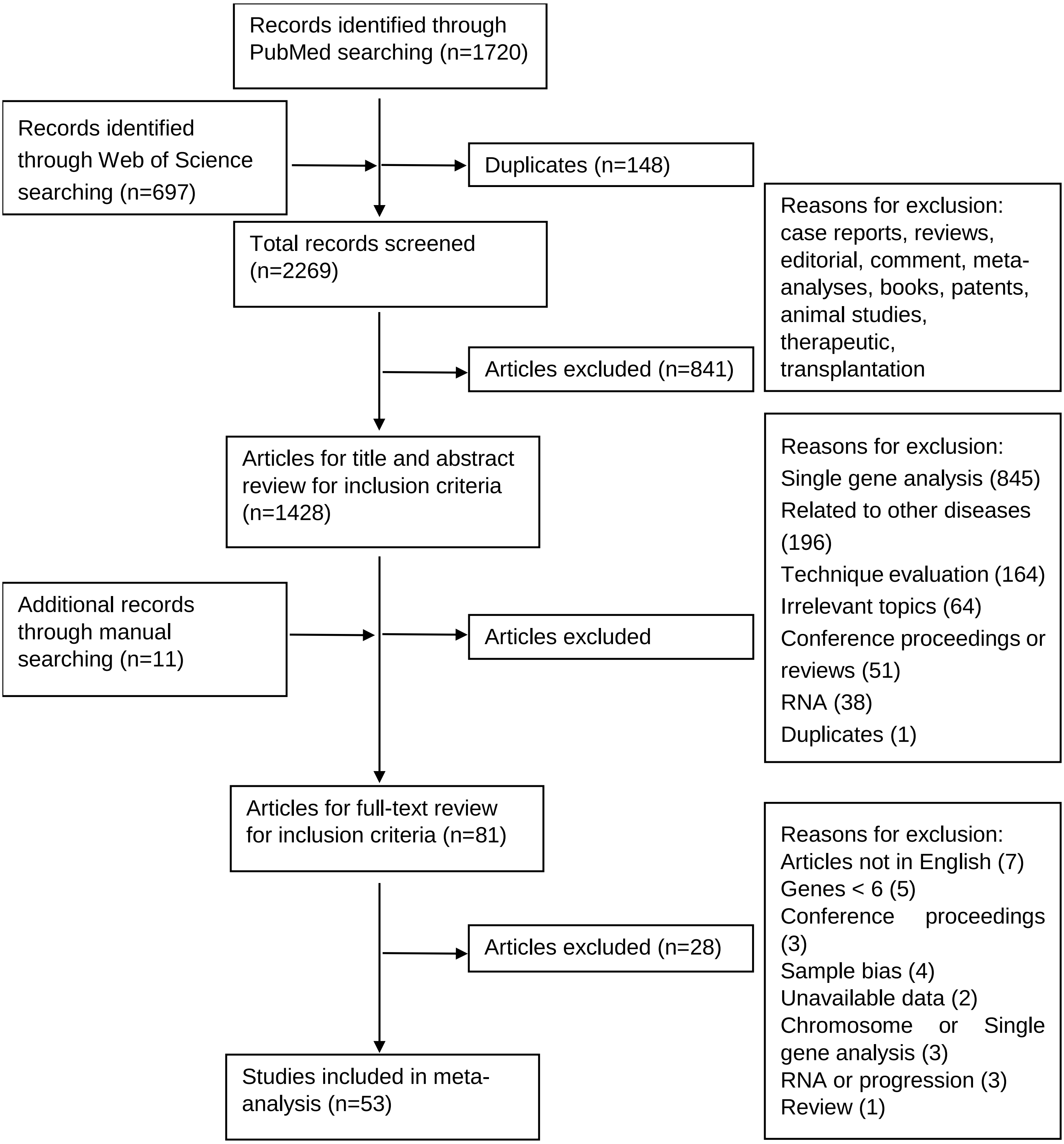

Figure S1. Flow chart eligibility criteria of articles included in meta-analysis. PubMed and Web of Science were selected for searching articles between January 2000 and March 2020. 2417 publications were identified by two databases and 148 duplicate studies were removed. The remaining 2269 articles were under the keyword assessment where 841 records in total were not qualified. 1428 articles from database searching and 11 studies from hand searching went further reviews of title and abstract. 1358 records were excluded according to the inclusion criteria and 81 articles were retrieved for full-text evaluation. Ultimately, 53 articles were included in meta-analysis after depth review.

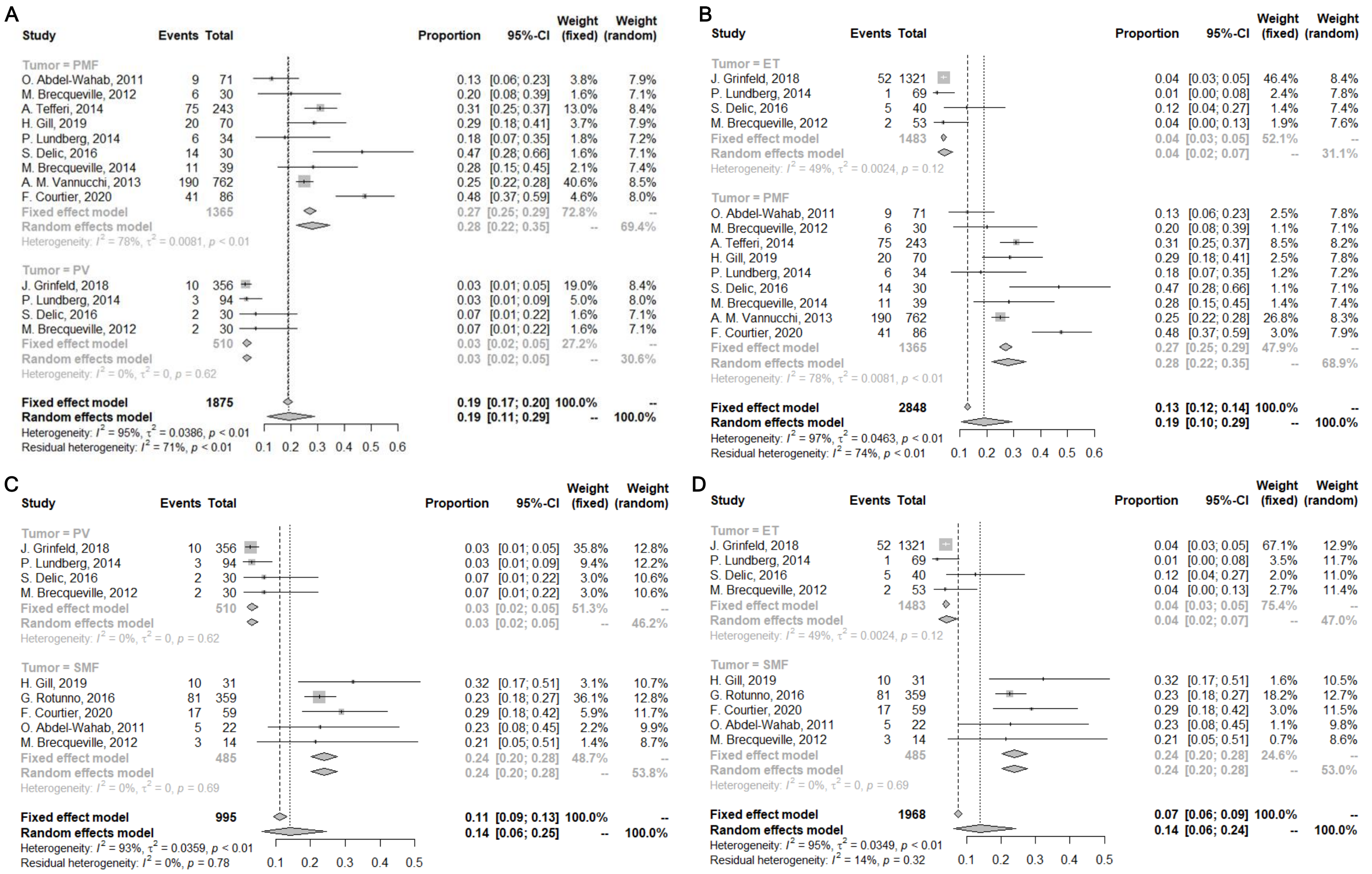

Figure S2. Forest plots of ASXL1 mutation, compared between (A) PMF and PV, (B) PMF and ET, (C) SMF and PV, (D) SMF and ET. Significant difference existed in all groups, p<0.0001.

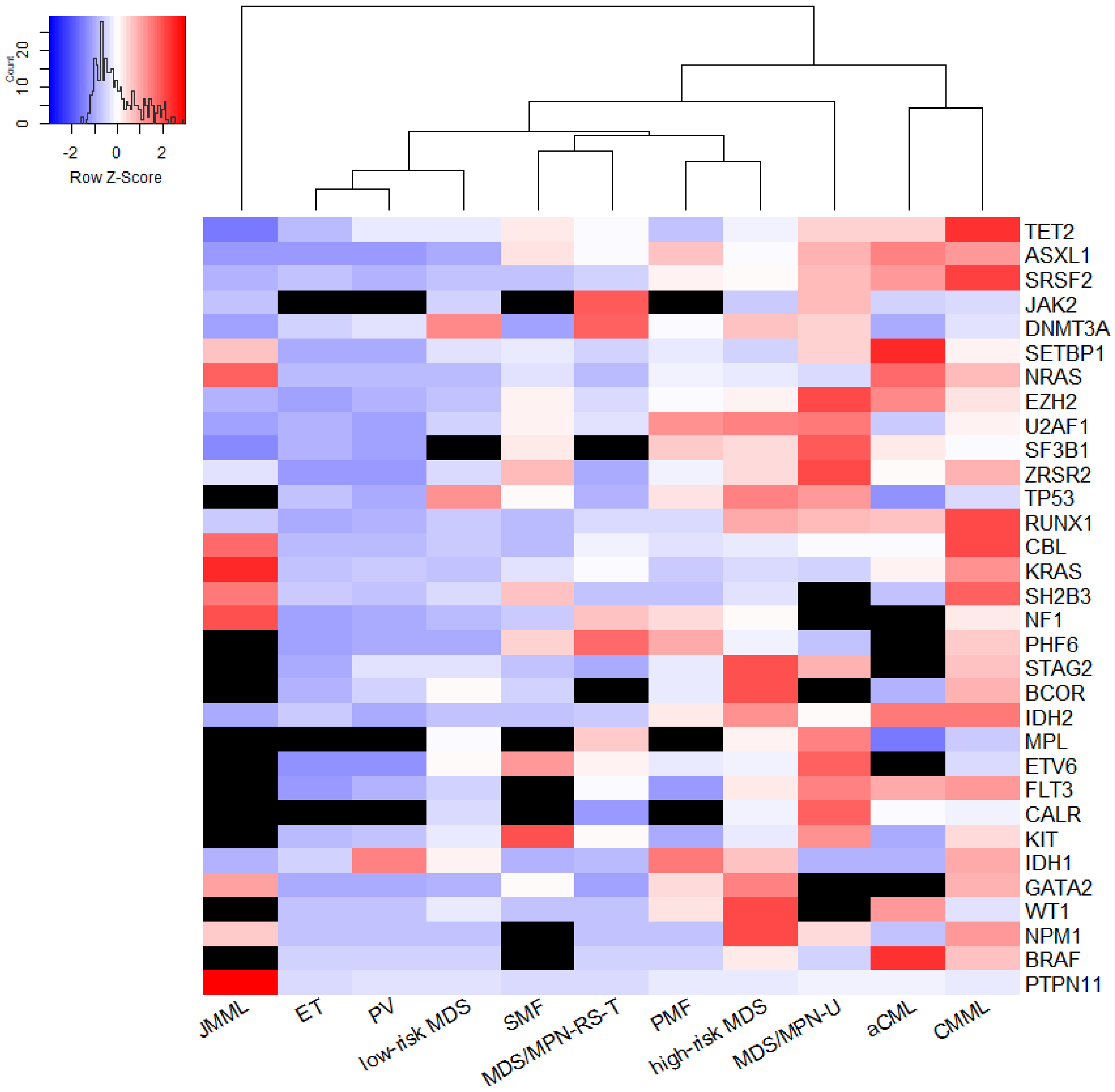

Figure S3. Heat map excluding the mutation rates of *JAK2, CALR and MPL* in MPN entities. ET and PV were similar to each other when three main driver genes were excluded, whereas PMF was shown to be distant from ET and PV, closer to MDS entities.
